# Ear Disorders among Aviators

**Published:** 1919-01

**Authors:** 


**Ear Disorders Among Aviators.** By M. André Castex. *Bulletin de l'Académie de Médecine.* June 25 1918.

The numerous medical inspections which I have made among aviators at the Villemin Military Hospital, and at the aviation center at Le Bourget have allowed me to analyze the ear disorders, the study of which is important in more ways than one.

It is usually at a height varying from 4,000 to 5,000 meters that aeroplanes have to fight. The barometer registers then from 47 to 41 atmospheres. The temperature is 1 degree lower per 110 meters in the lower altitudes, and 1 degree per 200 meters in the higher altitudes.

The disorders observed are :

1. *During the ascent :* A general fatigue due to the rapid diminution of the atmospheric pressure.

2. *During the flight proper :* At the altitude of 5,000 meters, pains in the ears, heaviness in the head, drowsiness, general lassitude and apathy.

3. *During the descent :* Pains in the ears and buzzing: all these disorders ceasing when he flies on the flat.

4. *On landing :* Transitory deafness and sometimes a rocking gait. Otoscopy reveals then that a congestion of the whole auditory apparatus has taken place.

In many cases a progressive diminution of the labyrinthic perception may be noticed.

It is also to be observed that there are latent alterations of the superior respiratory canals which are adjuvant causes of the deafness; — obstruction of the eustachian tube, a beginning of otosclerosis, hyperthrophic rhinitis, disformation of the partition, vegetations, adenoids, and enlarged tonsils.

These ear disorders are due especially to the difference of atmospheric pressure. It is why aviators are relieved if, when ascending, they resort to the Valsalva method, and, when descending, to Toynbee's.

Otiatrics is able to attenuate the disorders mentioned above.

